# Cellular pharmacology of fludarabine: molecular determinants of transport and metabolism

**DOI:** 10.3389/fphar.2026.1818501

**Published:** 2026-05-19

**Authors:** Mourad Mseddi, Vid Mlakar, Yvonne Gloor, Fanny Gonzales, Frederic Baleydier, Youssef Daali, Marc Ansari

**Affiliations:** 1 Cansearch Research Platform for Pediatric Oncology and Hematology, Department of Pediatrics, Gynecology, and Obstetrics, University of Geneva, Geneva, Switzerland; 2 Pediatric Onco-Hematology Unit, Department of Women, Child and Adolescent, University Geneva Hospitals, Geneva, Switzerland; 3 Clinical Pharmacology and Toxicology Division, University Hospitals of Geneva, Geneva, Switzerland

**Keywords:** drug resistance, fludarabine, metabolism, nucleoside transporter, personalized medicine, pharmacogenomics

## Abstract

Fludarabine monophosphate is an antimetabolite and a cornerstone of hematology and oncology treatments, with expanding clinical applications in hematological malignancies, stem cell transplantation conditioning, and chimeric antigen receptor T-cell (CAR-T) therapy. Despite widespread clinical use, there is substantial interpatient pharmacokinetic variability with up to 14.5-fold differences in drug exposure. Suboptimal exposures (both under- and overexposure) correlate directly with both treatment failure and non-relapse mortality, emphasizing the critical need for personalized dosing strategies. The therapeutic efficacy of fludarabine is determined by complex transport and metabolic processes. Cellular uptake is mediated primarily by human equilibrative (hENT1, hENT2) and concentrative nucleoside transporters (hCNT2, hCNT3). Once intracellular, deoxycytidine kinase catalyzes the rate-limiting phosphorylation step, converting fludarabine to its pharmacologically active triphosphate, which inhibits DNA synthesis and repair, ultimately driving cytotoxicity. The elimination mechanisms of fludarabine involve multiple pathways: cytoplasmic 5′-nucleotidase II and CD73-mediated dephosphorylation, while UDP-glucuronosyltransferases (particularly UGT2B17) catalyze glucuronidation-based elimination. The breast cancer resistance protein (BCRP/*ABCG2*) represents the principal efflux transporter, whereas fludarabine shows minimal interaction with P-glycoprotein and other major multidrug resistance-associated proteins. This review synthesizes current understanding of fludarabine’s cellular pharmacology, providing a framework for identifying biomarkers to guide personalized medicine approaches and to optimize fludarabine therapy and treatment outcomes.

## 1 Introduction

Fludarabine monophosphate (9-β-D-arabinofuranosyl-2-fluoroadenine-5′-monophosphate) is a fluorinated purine nucleoside analog that represents one of the key antimetabolites used in hematology; these also including cytarabine, nelarabine, clofarabine and cladribine (Lukenbill and Kalaycio, 2013; Kumamoto et al., 2020; Russo et al., 2005; Hermann et al., 2021; Andersson et al., 2011). Fludarabine is derived from purine nucleoside analog vidarabine, originally designed to resist adenosine deaminase (ADA)- mediated deamination (Brockman et al., 1977). Clinically, fludarabine has demonstrated significant efficacy in hematological malignancies such as chronic lymphocytic leukemia (CLL) and acute myeloid leukemia (AML) treatment, improving response rates and progression-free survival compared to previously used alkylating agents (Lukenbill and Kalaycio, 2013; Masood et al., 2011; Oliveira et al., 2023; Visani et al., 1994). It is widely used in conditioning regimens prior to hematopoietic stem cell transplantation (HSCT) in different myeloablative and non-myeloablative regimens and in lymphodepleting treatment for chimeric antigen receptor T-cell (CAR-T) therapy (Kumamoto et al., 2020; Myers et al., 2023; Harris et al., 2018; Ringdén et al., 2007; Mohanan et al., 2017; Talbot et al., 2014). Despite being in use for over 30 years, fludarabine is still administered with body surface area-based dosing protocols, although model-informed approaches are increasingly used in the HSCT and CAR-T cell settings (Keating et al., 1989; Brooks et al., 2022; Langenhorst et al., 2019a; Dekker et al., 2022; Nath et al., 2024; Ivaturi et al., 2017; Long-Boyle et al., 2011). As a consequence, substantial inter- and intrapatient exposure variability has been reported, with up to 14.5-fold variation in fludarabine area under the concentration-time curve (AUC) values (Mohanan et al., 2017; Langenhorst et al., 2019a; Ivaturi et al., 2017; Chung et al., 2019). Such variability in fludarabine exposure arises from differences in body size and renal function, as well as genetic variability and concomitant medications (Mohanan et al., 2017; Langenhorst et al., 2019a; Nath et al., 2024; Lichtman et al., 2002; Kemena et al., 1992). This marked pharmacokinetic (PK) heterogeneity can have significant clinical implications, as recent investigations have demonstrated clear exposure-response relationships in both HSCT conditioning and CAR-T cell therapy (Dekker et al., 2022; Kemena et al., 1992; Mikhael et al., 2022; Langenhorst et al., 2019b; Fabrizio et al., 2022; Nissani et al., 2021).

Understanding the PK and pharmacodynamics (PD) of this interindividual variability requires a close examination of the fludarabine molecular pathway. The clinical interest in fludarabine has highlighted critical knowledge gaps in our understanding of its molecular pharmacology. The last comprehensive review of fludarabine’s cellular and clinical pharmacology was published in 2002 by Gandhi and Plunkett, providing foundational insights into the drug’s mechanism of action, PK, and cellular PD (Gandhi and Plunkett, 2002). However, over the past 2 decades, we have witnessed remarkable advances in the understanding of nucleoside analog transport, metabolism, and resistance mechanisms, driven by sophisticated molecular biology techniques, high-resolution structural studies, and comprehensive pharmaco-omics analyses. The identification and characterization of specific nucleoside transporters, including human equilibrative nucleoside transporters (hENT1, hENT2) and human concentrative nucleoside transporters (hCNT1, hCNT2, hCNT3), have revealed the complexity of cellular fludarabine uptake mechanisms (Hediger et al., 2013; Pastor-Anglada et al., 2004; Molina-Arcas et al., 2005). Similarly, advances in understanding metabolic enzymes such as dCK, 5′-nucleotidases, and various efflux transporters have highlighted the interplay governing fludarabine uptake, activation and elimination (Shu et al., 2010; Yoshida et al., 2022; Minor et al., 2019; Walldén et al., 2007).

This review focuses on the cellular pharmacology of fludarabine, with particular emphasis on the transporters and enzymes that control its uptake, activation, inactivation and elimination. We aim to comprehensively characterize the pharmacological mechanisms that underpin variability in intracellular drug exposure and therapeutic response. These mechanistic insights may inform future strategies to optimize fludarabine-based regimens. Following an ADME-oriented scheme, the first part addresses influx mechanisms, detailing the transporters and selected additional carriers implicated in fludarabine entry into cells. The second part details intracellular metabolism, covering phosphorylation, dephosphorylation and other relevant enzymatic pathways that modulate the balance between active triphosphate and inactive metabolites. The final part discusses efflux mechanisms, focusing on ATP-binding cassette transporters and their variable contribution to fludarabine resistance compared with other nucleoside analogs.

## 2 General overview of fludarabine metabolism and mechanism of action

**FIGURE 1:**
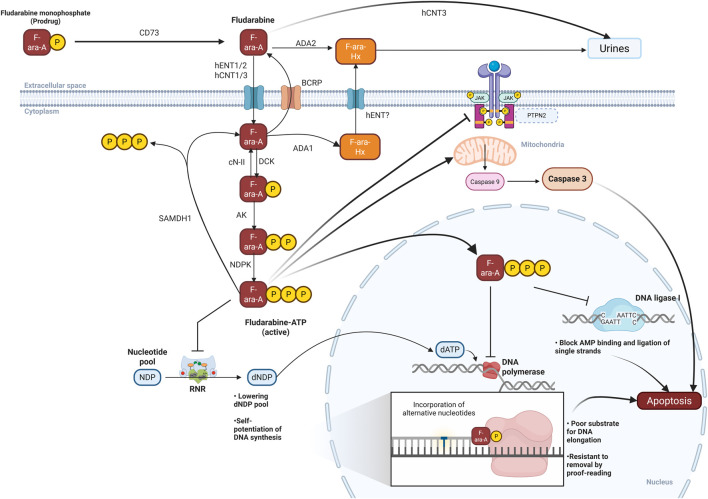
The metabolic pathway and mechanism of action of fludarabine. ADA, Adenosine deaminase; AK, Adenylate kinase; AMP, Adenosine monophosphate; BCRP, Breast cancer resistant protein; CD73, Ecto-5′-nucleotidase; cN-II, 5′-nucleotidase cytosolic II; dATP, Deoxyadenosine triphosphate; dCK, Deoxycytidine kinase; dNDP, Deoxyribonucleoside diphosphate; F-ara-A, Fludarabine; F-ara-Hx, Fludarabine hypoxanthine; hCNT1, human concentrative nucleoside transporter 1; hCNT3, human concentrative nucleoside transporter 3; hENT1, human equilibrative nucleoside transporter 1; hENT2, human equilibrative nucleoside transporter 2; JAK, Janus kinase; NDP, Nucleoside diphosphate; NDPK, Nucleoside diphosphate kinase; P, Phosphate; PTPN2, Protein tyrosine phosphatase non receptor type 2; RNR, Ribonucleotide reductase; SAMDH1, sterile alpha motif and histidine-aspartic domain-containing protein. Created in BioRender. Mlakar, V. (2026) https://BioRender.com/guqkye9.

The molecular basis of fludarabine’s therapeutic activity depends on several transport and metabolic processes (Figure 1). Following intravenous administration of the monophosphate prodrug, rapid dephosphorylation produces circulating nucleoside fludarabine, a highly hydrophilic compound that distributes predominantly within the vascular and extracellular spaces, and is transported across cell membranes by specialized nucleoside transporters (Lukenbill and Kalaycio, 2013; Gandhi and Plunkett, 2002; National Center for Biotechnology Information, 2026). Systematically, fludarabine is cleared mainly via the renal route (40%–60% in the first 24 h after IV administration), with hCNT3 mediating renal tubular uptake and resorption of fludarabine (Malspeis et al., 1990; Elwi et al., 2009a). Only a minor contribution from hepatic metabolism by UGT2B17 and UGT1A4 enzymes has been described (Allain et al., 2020a). Once in the cytoplasm, fludarabine undergoes sequential phosphorylation steps, primarily catalyzed by deoxycytidine kinase (dCK), to generate the pharmacologically active triphosphate metabolite–fludarabine triphosphate (fludarabine-ATP) (Gandhi and Plunkett, 2002). This active metabolite inhibits ribonucleotide reductase and DNA polymerase (Gandhi and Plunkett, 2002; Månsson et al., 1999). Additionally, it can be incorporated into DNA, ultimately triggering DNA strand termination and apoptosis preferentially in proliferating lymphoid cells (Huang and Plunkett, 1992).

## 3 Nucleoside-related transport mechanisms in the uptake of fludarabine

Cellular uptake of fludarabine is predominantly mediated by nucleoside transporters of the solute carrier (SLC) superfamily, mainly hENT (hENT1, hENT2) and hCNT (hCNT2, hCNT3), respectively (Figure 1). These proteins display overlapping specificities for natural nucleosides and related analogs cellular uptake (Hediger et al., 2013; Pastor-Anglada et al., 2004; Kaur et al., 2022). Their tissue-specific expression, described below, contributes to substantial cellular variability in fludarabine uptake, with lymphoblastic cells exhibiting 7-8 fold higher uptake than epithelial cells (Sirotnak and Barrueco, 1987; Stecula et al., 2017; Pastor-Anglada et al., 2022). In addition to the above-mentioned nucleoside transporters, the ergothioneine transporter (ETT) has also been proposed as a fludarabine carrier, but the evidence for its role in fludarabine uptake remains controversial (Müller and Gründemann, 2022; Drenberg et al., 2017; Buelow et al., 2021; Tschirka et al., 2018). Although phosphorylation is considered as the rate-limiting step in fludarabine cytotoxicity, altered transport mechanisms can modulate drug exposure and may contribute to resistance variability in specific cellular contexts (Månsson et al., 1999; Yamamoto et al., 2007).

### 3.1 hENTs

hENT1 and hENT2 are sodium-independent facilitative transporters encoded by *SLC29A1* and *SLC29A2*, broadly expressed in hematopoietic, epithelial, and endothelial cells (Pastor-Anglada et al., 2004; Kaur et al., 2022). Both mediate bidirectional transport of physiological nucleosides and several nucleotide analogs, including fludarabine. hENT2 additionally transports nucleobases such as hypoxanthine and shows relative insensitivity to the classical hENT1 inhibitor nitrobenzylmercaptopurine riboside (NBMPR) (Molina-Arcas et al., 2005; Griffiths et al., 1997). Although hENT1 appears to be the dominant hENT transporter for fludarabine, experimental data support a non-negligible role of hENT2 in fludarabine uptake.

Functionally, hENT1 has been firmly linked to fludarabine uptake and cytotoxicity. In a fludarabine-resistant T-cell acute lymphoblastic leukemic (ALL) cell line (CEM) that is transport-deficient, fludarabine potency is reduced threefold compared with hENT1-expressing counterparts (King et al., 2006). In a separate experiment, nelarabine-resistant CEM cells show decreased hENT1 transcription in parallel with the cross-resistance to other nucleoside analogs (Yamauchi et al., 2014).

hENT1 mRNA levels from 22 fludarabine-treated CLL patients showed no association with *ex-vivo* drug sensitivity or clinical outcome, whereas hENT-mediated fludarabine transport activity significantly correlates with cell viability, underscoring the limited value of transcript quantification as a biomarker (Woodahl et al., 2009; Mackey et al., 2005; Molina-Arcas et al., 2003). hENT2 protein expression, rather than mRNA, has been associated with fludarabine uptake and cytotoxicity in CLL, further highlighting the importance of protein activity (Molina-Arcas et al., 2005; Woodahl et al., 2009; Mackey et al., 2005; Ward et al., 2003; López-Guerra et al., 2008). Notably, the clinical importance of hENTs expression appears to be context-dependent. In AML patients treated with cytarabine, low hENT1 mRNA expression at diagnosis is associated with increased risk of early relapse and inferior survival, supporting a prognostic role for hENT1 in this setting, while similar associations for fludarabine in CLL have not been demonstrated (Mackey et al., 2005; Galmarini et al., 2002).

### 3.2 hCNTs

hCNT2 and hCNT3 are sodium-dependent, active transporters, encoded by *SLC28A2* and *SLC28A3,* respectively, with prominent expression in renal, intestinal, and subsets of leukemic cells (Pastor-Anglada et al., 2004; Kaur et al., 2022). hCNT3 exhibits broad purine/pyrimidine specificity and forms homotrimeric complexes that mediate high-affinity concentrative uptake of purine analogs, including fludarabine, whereas hCNT2 preferentially transports purine nucleosides (also accepting uridine) and displays low-rate fludarabine uptake (Stecula et al., 2017; Pastor-Anglada et al., 2022; King et al., 2006; Lang et al., 2004).

*In vitro* studies consistently identify hCNT3 as a major transporter of fludarabine when present at the plasma membrane. In human renal proximal tubular cells (hRPTC), fludarabine uptake correlates significantly with hCNT3 surface protein abundance, and oocytes expressing hCNT3 demonstrate higher fludarabine accumulation than controls (Elwi et al., 2009a; Ritzel et al., 2001). In CLL cells with the major TP53 allele, which are highly sensitive to fludarabine, basal hCNT3-dependent uptake is prominent, whereas resistant cells rely primarily on ENT-mediated transport, with negligible sodium-coupled hCNT3 activity (Fernández-Calotti et al., 2012). Experiments with transfected oocytes showed that hCNT2 provides a minor uptake route for cladribine, compared with hCNT3 and hENTs, which together account for the bulk of transport (King et al., 2006).

As for hENTs, hCNT2 and hCNT3 mRNA levels do not correlate with *in vitro* fludarabine sensitivity, fludarabine-ATP formation, or clinical response in CLL, suggesting considerable post-transcriptional regulation (Molina-Arcas et al., 2005; Elwi et al., 2009a; Woodahl et al., 2009; López-Guerra et al., 2008). In hRPTC, large variability in hCNT3 mRNA expression also failed to predict cell surface protein levels or transporter activity. On the other hand, in CLL, high hCNT3 transcript levels have paradoxically been associated with poor response to fludarabine, explained by immunohistochemical evidence of intracellular, rather than membranous, protein localization (Elwi et al., 2009a; Mackey et al., 2005). However, blast-level expression data for SLC28A3 in acute leukemias are limited, and there is currently no direct evidence of a similar role for hCNT3 in ALL blasts, whereas in AML, high hCNT3 has been documented in t (8; 21)+ cases and is associated with better response and survival following cytarabine-based chemotherapy (Song et al., 2015).

These observations collectively indicate that hCNT3 is a functionally important transporter for fludarabine when correctly trafficked to the cell surface and that transcript abundances of all transporters (hENTs and hCNTs) may not be accurate surrogates for active transport capacity.

### 3.3 Ergothioneine transporter (ETT)

In contrast to *SLC29A* and *SLC28* nucleoside transporters, the ETT (formerly known as OCTN1) belongs to the *SLC22A4* family, which is not considered a canonical nucleoside carrier (Galluccio et al., 2024; Gründemann et al., 2022). ETT is a sodium-dependent transporter with high affinity for the amino acid ergothioneine, which is highly expressed in bone marrow, erythroid precursors, monocytes, kidney, and intestinal tissues (Galluccio et al., 2024; Gründemann et al., 2005).

Initial studies suggested that ETT could transport several nucleoside analogs, including fludarabine, with greater apparent efficiency than hENT1, and that higher *SLC22A4* expression in pediatric AML is associated with improved survival after nucleoside-based chemotherapy (Drenberg et al., 2017). Epigenetic silencing of *SLC22A4* by promoter methylation has been proposed as a resistance mechanism to nucleoside analogs (Buelow et al., 2021). However, subsequent studies could not reproduce nucleoside transport by ETT, suggesting that earlier findings may reflect off-target effects or activity of other co-expressed transporters (Müller and Gründemann, 2022; Tschirka et al., 2018).

Overall, current evidence regarding ETT as a potential pharmacologically relevant transporter remains inconclusive and warrants further investigations.

## 4 Nucleoside-related metabolism of fludarabine

Like most anticancer or antiviral nucleoside analogs, fludarabine needs to undergo a series of phosphorylation steps before its incorporation into the DNA.

While fludarabine can enter cells through multiple transporters, current evidence indicates that metabolism via phosphorylation, dephosphorylation, and deamination plays a more decisive role in determining its cytotoxic activity and resistance profile than the route of entry (Elwi et al., 2009a; Drenberg et al., 2017; Yamauchi et al., 2014; Mackey et al., 2005; Galmarini et al., 2002; Ritzel et al., 2001; Song et al., 2015) (Figure 1).

### 4.1 Fludarabine phosphorylation by dCK

Cellular phosphorylation of fludarabine, primarily mediated by dCK, is essential for its efficacy in cancer therapy (Månsson et al., 1999). dCK is a rate-limiting enzyme in the deoxyribonucleoside salvage pathway (Arnér and Eriksson, 1995). It phosphorylates all three natural deoxyribonucleosides (deoxycytidine, deoxyadenosine and deoxyguanosine), as well as several nucleoside analogs, including fludarabine, cytarabine, cladribine, clofarabine, gemcitabine, decitabine or nelarabine and several antiviral agents (Yoshida et al., 2022; Arnér and Eriksson, 1995; Wong et al., 2009; Stegmann et al., 1995; Eriksson et al., 1991; Sabini et al., 2007; Fi et al., 2018). It uses preferentially UTP, ATP, or other nucleoside triphosphates as phosphate donors and displays the highest affinity for deoxycytidine and lower affinities for purine deoxynucleosides and their analogs (Eriksson et al., 2002). The enzyme is regulated through feedback inhibition by cytidine triphosphate, and all of its substrates serve as competitive inhibitors (Kemena et al., 1992; Avramis et al., 1989).

dCK is a cytoplasmic homodimer (≈30 kDa subunits) encoded by a GC-rich gene on chromosome 4, with seven exons and a promoter lacking TATA or CAAT boxes but containing multiple transcription factor binding sites (Arnér and Eriksson, 1995; Song et al., 1993; Stegmann et al., 1993). Highest expression and activity are found in lymphoid tissues and lymphocytes, whereas levels are very low in differentiated parenchymal cells of organs such as liver, muscles, kidney and pancreas (Shu et al., 2010; Eriksson et al., 2002; Radu et al., 2008; Eriksson et al., 1994; Spasokoukotskaja et al., 1995). *In situ* immunofluorescence studies showed selective dCK accumulation in lymphoid organs and predominantly perinuclear cytoplasmic localization, explaining the lymphodepleting properties of fludarabine (Hatzis et al., 1998).

Functionally, dCK activity levels vary significantly across cancer cell types. Leukemic cells (e.g., ALL cell line L1210 and chronic myelogenous leukemia K562), exhibit higher fludarabine phosphorylation rates than epithelial cells or resistant cells to nucleoside analogs (Sirotnak and Barrueco, 1987; Hapke et al., 1996). In cytarabine-resistant cells, the phosphorylation of fludarabine is reduced by 16-fold compared to non-resistant cells (Brockman et al., 1980). Biochemical assays revealed that dCK phosphorylates 2-fluoroadenine derivatives at a faster rate than natural substrates, and its apparent substrate affinity depends on the phosphate donor, with UTP supporting higher phosphorylation efficiency rates than ATP or mixed nucleotides (Tseng et al., 1982; Shewach et al., 1992). Competition between nucleoside analogs is clinically relevant: cytarabine acts as a competitive inhibitor of fludarabine phosphorylation and fludarabine-ATP accumulation, both *in vitro* and in CLL patients, where the sequential administration profoundly affects fludarabine PK (Kemena et al., 1992). Conversely, in AML, fludarabine is commonly combined with high-dose cytarabine in FLAG (fludarabine, cytarabine, and G-CSF) regimens, where fludarabine administration beforehand leads to an increasing intracellular cytarabine triphosphate formation and enhances its antileukemic activity (Visani et al., 1994; Gandhi et al., 1993). Beyond its interaction with cytarabine, fludarabine has also been combined with the second-generation deoxyadenosine analog clofarabine in a conditioning regimen. Preclinical data show synergistic cytotoxicity when fludarabine and clofarabine are combined with busulfan, leading to increased DNA damage signaling and apoptosis in leukemic blasts (Valdez et al., 2011). Clinically, the association of clofarabine, fludarabine and busulfan has produced encouraging outcomes in adults and children with high-risk ALL and AML and is increasingly adopted as a standard reduced-toxicity conditioning (Andersson et al., 2011; Versluijs et al., 2022).

Loss or reduction of dCK activity is the most consistent molecular marker of resistance to fludarabine and other related analogs (Månsson et al., 1999; Yamamoto et al., 2007; Dumontet et al., 1999; Bai et al., 1998; Klanova et al., 2014). Resistant cell lines frequently harbor dCK deletions or mutations (Månsson et al., 1999; Yamamoto et al., 2007; Hapke et al., 1996; Dumontet et al., 1999; Rivero et al., 2011). Multiple studies have demonstrated that resistant cells carried gene deletions or other mutations resulting in significant reductions in dCK activity (Månsson et al., 1999; Yamamoto et al., 2007; Dumontet et al., 1999). Lower dCK activity decreases intracellular accumulation of fludarabine-ATP, thereby impairing fludarabine efficacy, and correlates with cross-resistance to other nucleoside analogs such as cytarabine, cladribine, and gemcitabine (Hapke et al., 1996; Dumontet et al., 1999; Bai et al., 1998; Klanova et al., 2014). Importantly, preserved nucleoside transporter activity can not overcome impaired phosphorylation, as some fludarabine-resistant cells can maintain fludarabine uptake yet still accumulate less active triphosphate (Bai et al., 1998).

In addition, genetic polymorphisms in *dCK* have been associated with clinical outcomes and toxicities. In a study on patients treated with fludarabine-based regimens for follicular lymphoma, *dCK* variants −360C>G, −201C>T, C28624T, c.91 + 37G>C and −12C>G were evaluated for association with lower hematologic toxicity. Only C28624T showed a statistically significant relationship with a lower risk of lymphopenia (Rivero et al., 2011). However, dCK mRNA expression in healthy donors did not differ significantly between variant and wild-type dCK carrier patients (Rivero et al., 2011). dCK transcript levels were not associated with *in vitro* resistance to fludarabine or with fludarabine triphosphate accumulation in primary CLL patients’ cells (Woodahl et al., 2009; López-Guerra et al., 2008).

Post-transcriptional regulation is central for dCK activity. Enzyme activity can vary 10- to 15-fold across the cell cycle, while the mRNA levels remain stable, suggesting a dynamic regulation shown to be controlled by phosphorylation and protein-protein interactions (McSorley et al., 2008; Bunimovich et al., 2014; Beyaert et al., 2017; Hazra et al., 2011; Hengstschläger et al., 1993; Wang et al., 2025). Ataxia telangiectasia mutated kinase (ATM) and ATM Rad3 related (ATR) dependent phosphorylation at Serine 74 (Ser-74) increases dCK activity in response to DNA damage (McSorley et al., 2008; Bunimovich et al., 2014; Beyaert et al., 2017; Hazra et al., 2011). Interaction with proteins such as cyclin-dependent kinase 1 (Cdk1) and HSP90 modulates its stability and function within cell-cycle checkpoints (Hengstschläger et al., 1993; Wang et al., 2025). Site-specific modifications of dCK, such as the phosphorylation at Ser-74, modulate kinetic rates in a substrate-dependent manner. For fludarabine, the Ser-74 mutation increases kcat with UTP but markedly lowers catalytic efficiency, suggesting that these regulatory effects are substrate-selective (Amsailale et al., 2012). In addition, dCK activity is dynamically regulated by exposure to nucleoside analogs. For example, exposure to cladribine or fludarabine in S-phase cells can markedly increase enzyme activity (Spasokoukotskaja et al., 1999). These findings exemplify the complex activity of dCK for therapeutic response (Woodahl et al., 2009; López-Guerra et al., 2008; Rivero et al., 2011; Spasokoukotskaja et al., 1999).

Although mitochondrial deoxyguanosine kinase (dGK) can also phosphorylate fludarabine and related analogs, its contribution is minor compared to dCK (Månsson et al., 1999; Yamauchi et al., 2014; Spasokoukotskaja et al., 1999; Sjöberg et al., 1998).

Overall, fludarabine phosphorylation by dCK emerges as a key determinant of pharmacologic efficacy and toxicity, with resistance driven by genetic polymorphisms, downregulation, and post-translational modulation that impair drug activation, supporting the rationale for integrating dCK activity status into biomarker-guided individualized fludarabine therapy.

### 4.2 Mechanisms of fludarabine dephosphorylation

Dephosphorylation of fludarabine nucleotides is a key inactivation pathway that counteracts the formation and persistence of the active metabolite fludarabine-ATP. This process is mainly mediated by 5′-nucleotidases, which convert phosphorylated metabolites back to the parent nucleoside (Figure 1). Collectively, data indicate that 5′-nucleotidases cooperate to dephosphorylate fludarabine nucleotides at intra- and extracellular sites, and that increased 5′-nucleotidase activity—especially when combined with reduced dCK—can shift the balance towards drug inactivation and contribute to clinical resistance.

#### 4.2.1 Fludarabine dephosphorylation by cytoplasmic 5′-nucleotidase II (cN-II)

cN-II is encoded by the *NT5C2* gene on chromosome 10 and produces a 561–amino acid cytosolic protein of approximately 65 kDa (Gene: NT5C2, 2026). cN-II is a ubiquitous, tetrameric enzyme of the haloacid dehalogenase superfamily that hydrolyses purine and purine-analog monophosphates, including fludarabine monophosphate, back to their corresponding nucleosides (Walldén et al., 2007; Ipata and Tozzi, 2006; Jordheim et al., 2006; Gallier et al., 2011).

Work on enzyme kinetics by Jordheim et al. demonstrated that fludarabine monophosphate is a direct substrate of cN-II (Km ≈ 10 μM, Vmax ≈35 nmol/min/mg), confirming its capacity to efficiently dephosphorylate fludarabine monophosphate (Jordheim et al., 2006). Functionally, overexpression of cN-II is a well-described *in vitro* resistance mechanism to purine analogs. In HEK293 cells, Cividini et al. showed that cN-II overexpression increased the half maximum inhibitory concentration (IC50) of fludarabine by ∼82-fold compared with control cells, together with profound alterations of nucleotide pools, demonstrating that enhanced dephosphorylation can almost abolish cytotoxicity despite intact upstream activation (Cividini et al., 2015). Consistent with this, differences in cN-II expression modulate T-cell sensitivity: CD8^+^ lymphocytes, which have lower cN-II mRNA and higher dCK/cN-II ratios, display greater susceptibility to fludarabine-induced apoptosis than CD4^+^ cells (Gam et al., 2003). However, transcript-based analyses in HSCT cohorts did not consistently correlate cN-II mRNA with fludarabine-ATP accumulation (Yamauchi et al., 2014; Woodahl et al., 2009).

#### 4.2.2 Fludarabine dephosphorylation by ecto-5′-nucleotidase

Ecto-5′-nucleotidase (CD73), encoded by *NT5E* located on chromosome 6, is a GPI- cell surface enzyme that dephosphorylates extracellular purine and pyrimidine nucleoside monophosphates to nucleosides and contributes to purinergic homeostasis in many tissues (Minor et al., 2019; Colgan et al., 2006; Sträter, 2006). CD73 is widely expressed on epithelial and endothelial cells, fibroblasts and lymphocyte subsets; its deficiency causes arterial calcification, underscoring its physiological relevance (Minor et al., 2019; Resta et al., 1998). In the tumor microenvironment, CD73 further exerts a potent immunosuppressive effect by generating adenosine, which signals to dampen T- and NK-cell immune response (Allard et al., 2012).

In the setting of nucleoside-based chemotherapy, higher CD73 expression has been associated with unfavorable outcomes. In AML patients treated with cytarabine, Galmarini et al. reported that the presence of CD73-positive blasts at diagnosis was associated with shorter disease-free survival (DFS) (11 vs. 19.8 months, p = 0.007) and overall survival (OS) (14 vs. 30 months, p = 0.003), and multivariate analysis identified CD73 positivity as conferring a 3.6-fold higher relapse risk (p = 0.0001), supporting a role for ecto-5′-nucleotidase activity in resistance (Galmarini et al., 2002). In CLL patients receiving fludarabine, Mackey et al. observed that high CD73 mRNA expression correlated with a markedly shorter time to progression (257 days vs. 777 days, adjusted HR = 0.32, p = 0.022) (Mackey et al., 2005). Additionally, a pharmacogenetic study in HSCT patients found that a promoter polymorphism in *NT5E* (*rs2295890*) was associated with a ∼29% reduction in fludarabine clearance in variant carriers (p = 0.038) (Mohanan et al., 2017). Studies indicate that a balance between activating and inactivating enzymes determines cellular sensitivity to fludarabine (Månsson et al., 1999; Dumontet et al., 1999). For example, Dumontet et al. demonstrated that fludarabine-resistant K562 erythroleukemic cells showed increased total 5′-nucleotidase activity and decreased deoxycytidine kinase (dCK) activity, resulting in lower levels of therapeutic triphosphate metabolites (Dumontet et al., 1999).

#### 4.2.3 Subsequent phosphorylation/dephosphorylation steps (AK, NDPK, CMPK1)

By analogy to natural nucleoside monophosphates, the downstream phosphorylation steps from fludarabine monophosphate to diphosphate and triphosphate are attributed to involve adenylate kinase (AK) and nucleoside diphosphate kinase (NDPK), although direct experimental evidence with fludarabine is limited (Figure 1).

AK enzymes are ubiquitous enzymes that maintain cellular adenine nucleotide balance by catalyzing the reversible transfer of the γ-phosphate group from ATP to AMP (AMP + ATP ⇌ 2 ADP). The AK family comprises nine isoforms with 15%–60% sequence homology and a shared preference for AMP as the main substrate (Dzeja and Terzic, 2009). To date, no study has directly tested AK-mediated phosphorylation of fludarabine or linked AK polymorphisms to fludarabine efficacy or toxicity, but *in vitro* work has shown that several AK2 variants (V19G, K28R, K62E, T194I) reduce phosphorylation of the nucleotide analog tenofovir, suggesting that AK genetic variation could, by extension, modulate activation of other nucleoside analogs (Fi et al., 2018).

Similarly, nucleoside diphosphate kinases (NDPK, encoded by *NME1/2*) are presumed to catalyze the final step from fludarabine diphosphate to active triphosphate. NDPKs are highly conserved enzymes and exhibit broad substrate specificity, accepting both purine and pyrimidine substrates (Schaertl et al., 1998). Although direct biochemical data with fludarabine are lacking, NDPK1/2 have been shown to catalyze the last phosphorylation step of cytarabine and other nucleoside analogs (Braunagel et al., 2012). Polymorphisms in *NME1* (e.g., *rs2302254*) have been associated with treatment-related neurotoxicity without reaching statistical significance, whereas *NME2 (rs3744660)* variant has been linked to lower complete response (CR) rates in cytarabine-treated AML (OR = 1.83; p = 0.03 in homozygous and heterozygous variant carriers) (Braunagel et al., 2012; Zhu et al., 2018). Interpretation is complicated by reports that high *NME1* expression correlates with poor prognosis in AML and that NDPK2 also exhibits tumour-suppressor properties (Postel et al., 1993; Okabe-Kado et al., 1998).

Cytidine monophosphate kinase 1 (CMPK1), also known as UMP/CMP kinase 1, plays a crucial role in the metabolism of pyrimidine analogs such as cytarabine and gemcitabine monophosphates, converting them to their diphosphate forms (Ryu et al., 2011). CMPK1-reduced protein expression was associated with cell resistance to pyrimidine nucleoside analogs, but not to purine analogs, such as fludarabine (Topalis et al., 2016). Still, there is no direct experimental evidence that CMPK1 is significantly involved in fludarabine metabolism.

#### 4.2.4 Fludarabine-ATP dephosphorylation by sterile alpha motif and histidine-aspartic domain-containing protein 1 (SAMHD1)

SAMHD1 is a 626-amino acid protein, encoded by the *SAMHD1* gene on chromosome 20, initially identified as part of the human innate immune system and highly expressed in monocytes, macrophages, dendritic cells, and resting T cells (Johansson et al., 2018; Knecht et al., 2018). It localizes predominantly to the nucleus and exhibits cell-cycle–dependent expression, with the highest levels in quiescent cells and a marked decline during S-phase (Franzolin et al., 2013). Beyond its role in innate immunity, SAMDH1 acts as a deoxynucleoside triphosphatohydrolase (dNTPase), hydrolyzing dNTPs and nucleoside analog triphosphates, such as fludarabine-ATP, into nucleoside and inorganic triphosphates, thereby decreasing the intracellular pool of active metabolites and contributing to genome stability (Knecht et al., 2018; Rossi, 2014) (Figure 1).

SAMHD1 has emerged as a pharmacologically relevant modulator of nucleoside-analog response. In antiretroviral therapy, SAMHD1 enhances the efficacy of several nucleoside reverse-transcriptase inhibitors (NRTI) by depleting intracellular dNTPs and favoring incorporation of thymidine-analog triphosphates into viral DNA (Lahouassa et al., 2012). In AML, SAMDH1 expression levels accurately predict therapeutic responses to cytarabine; deleting SAMHD1, achieved by genetic depletion, mutational inactivation and proteasomal degradation using virus-like particles, increases cytarabine sensitivity and cytotoxicity in leukemia cells and mouse models (Schneider et al., 2017). Moreover, the SAMHD1 eQTL polymorphism *rs6102991*, which is associated with reduced enzyme activity, was found to be associated with a decreased risk of non-achieving CR in 361 AML patients receiving cytarabine (p = 0.055) (Zhu et al., 2018). However, the clinical impact of SAMHD1 appears to be context- and drug-dependent. In 189 mantle cell lymphoma patients treated with cytarabine or fludarabine, high SAMHD1 expression does not significantly affect CR rates or event-free survival (Roider et al., 2021). In T-cell prolymphocytic leukaemia, SAMHD1 mutations do not clearly restore sensitivity to fludarabine or other nucleoside analogs (Johansson et al., 2018).

Collectively, evidence suggests a role for SAMHD1 in fludarabine metabolism, but further research is necessary to elucidate the specific mechanisms underlying this activity and to determine SAMHD1’s role in treatment response.

### 4.3 Fludarabine deamination by ADA

ADA is a key circulating enzyme in purine metabolism. Physiologically, it catalyzes the irreversible deamination of adenosine and deoxyadenosine to inosine and deoxyinosine, therefore regulating intracellular and extracellular concentrations of adenosine (Bagheri et al., 2019).

The human *ADA* gene is located on chromosome 20 and is mainly expressed in the lymphoid organs, such as lymph nodes, spleen, and thymus (Paul et al., 2017; Valerio et al., 1985). It plays an essential role in immune cell development and differentiation (Adams and Harkness, 1976; Chinsky et al., 1989). Under normal physiological conditions, ADA1 exists mainly as an intracellular monomeric enzyme but can also form dimeric complexes, while ADA2 is a distinct isoform produced mostly by monocyte-macrophage lineage and circulates as a homodimer in serum (Costa et al., 2021). The broad tissue distribution of ADA and its interaction with surface molecules like CD26 enable it to regulate both intra- and extracellular adenosine levels, orchestrating immune and metabolic homeostasis (Gonzalez-Gronow et al., 2004; Moreno et al., 2018). ADA deficiency is the primary cause of autosomal recessive severe combined immunodeficiency (SCID), with ADA loss leading to accumulation of deoxyadenosine and deoxyadenosine triphosphate in developing lymphocytes, resulting in lymphocyte apoptosis and compromised T- and B-cell immunity (Bradford et al., 2017; Flinn et al., 2018).

Fludarabine was originally designed to avoid the conversion by ADA, and several investigations have supported this view. In cell culture studies, the inhibition of ADA by deoxycoformycin did not affect fludarabine-ATP accumulation (Avramis and Plunkett, 1983). Similarly, Brockman et al. found that the intracellular levels of fludarabine-ATP in L1210 cells treated with fludarabine were comparable to the levels of the active metabolite of cytarabine, arabinofuranosyladenine triphosphate (ara-ATP), in cells treated with cytarabine in the presence of an ADA inhibitor (Brockman et al., 1977). Despite its relative resistance, fludarabine is nevertheless subject to ADA-mediated metabolism *in vivo* (Figure 1). Noker et al. observed that fludarabine is deaminated to fludarabine-hypoxanthine (fludarabine-Hx) following administration of either 40 mg/m^2^ or 500 mg/m^2^ in mice and dogs. In dogs, approximately equal percentages of fludarabine and fludarabine-Hx were recovered in urine at both dosing levels (Noker et al., 1983). Consistently, in patients with CLL who received a 25 mg/m^2^ IV infusion over 30 min, fludarabine-Hx was detected, with AUC representing 20% of the total fludarabine AUC and urinary excretion of 13% ± 6% of the administered dose (Yin et al., 2010). These findings highlight that although fludarabine is designed to resist ADA deamination, the enzyme still contributes to its metabolic clearance *in vivo*. This balance between resistance and partial metabolism is crucial for understanding fludarabine’s pharmacological activity and therapy optimization.

## 5 Fludarabine glucuronidation by UGT

The glucuronidation of fludarabine is primarily catalyzed by two specific UGT enzymes: UGT2B17 and UGT1A4. These enzymes represent the main conjugating mechanism responsible for fludarabine inactivation via phase II metabolism, both demonstrating the capacity to form two glucuronide metabolites (G1 and G2), with G2 forming preferentially over G1 (Allain et al., 2020a). Importantly, this study only demonstrated the presence of fludarabine-glucuronide without formally quantifying its relative abundance to the parent drug. The reported plasma concentrations of the metabolite were low, indicating a minor contribution of this pathway to overall clearance (Allain et al., 2020a).

Importantly, this study only demonstrated the presence of fludarabine-glucuronide, without formally quantifying it relative to the parent drug. The plasma concentrations of the metabolite were low, indicating a minor role for this pathway in overall clearance (Malspeis et al., 1990).

UGT2B17 is particularly significant in CLL due to its predominant expression in lymphoid tissues, while other UGT family members remain at very low or undetectable levels (Gruber et al., 2013; Allain et al., 2020b). In CLL patients who exhibit UGT2B17 induction after fludarabine treatment, basal UGT2B17 expression is 7.5-fold higher compared to those without induction. This induction occurs rapidly, being detected within hours to days after treatment initiation and is particularly pronounced in patients who fail to respond to therapy (Allain et al., 2020a). Consistently, increased UGT2B17 expression is associated with poor prognosis in CLL patients after receiving fludarabine-based regimens, with higher UGT2B17 expression associated with shorter treatment-free (HR = 2.25; 95% CI 1.58–3.27) and OS (HR = 2.18; 95% CI 1.18–4.01) (Gruber et al., 2013). While UGT1A4 has significant fludarabine glucuronidation activity, it differs from UGT2B17 in several aspects. Unlike UGT2B17, UGT1A4 is responsible for the conjugation of a broad range of molecules, such as BTK/PI3K inhibitors (Allain et al., 2020a). UGT1A4 is much less abundant than UGT2B17 in leukemic B cells (often undetectable at mRNA levels in CLL patients before treatment with a fludarabine-based regimen). However, UGT1A4 is highly expressed in the liver and in the bile ducts, colon and small intestines and is likely responsible for hepatic conjugation (Allain et al., 2020a; Benoit-Biancamano et al., 2009). Altogether, this suggests that the UGT pathway participates in fludarabine inactivation and could be a mechanism of fludarabine clearance (Allain et al., 2020a; Gruber et al., 2013).

## 6 Mechanisms of efflux transport of fludarabine

ATP-binding cassette (ABC) transporters are important determinants of intracellular drug export and, therefore, are often responsible for multidrug resistance. However, their impact on fludarabine is heterogeneous and generally less pronounced than for many other cytotoxic agents (Pote and Gacche, 2023; Fukuda and Schuetz, 2012). Among them, breast cancer resistance protein (BCRP/*ABCG2*) shows a clearer association with resistance, whereas P-glycoprotein (P-gp/*ABCB1*) appears to have limited relevance for fludarabine, and multidrug resistance–associated proteins (MRPs/*ABCC*s) mainly affect other nucleoside analogs with only indirect or genetic evidence for fludarabine.

### 6.1 BCRP

BCRP is encoded by *ABCG2* and highly expressed in intestinal epithelium, hepatocytes, renal proximal tubules, placenta and the blood–brain barrier, where it limits xenobiotic absorption and tissue penetration of drugs (Robey et al., 2009; Doyle et al., 1998). BCRP overexpression has been more directly linked to fludarabine resistance in both clinical and experimental settings.

In a cohort of 138 adult AML patients undergoing fludarabine-based induction therapy, BCRP protein overexpression was associated with higher relapse rates (44.5% vs. 28.5%, p = 0.04) and earlier relapse (log-rank test = 6.02, p = 0.04) (Damiani et al., 2010). At a cellular level, MDCKII kidney cells overexpressing human or mouse *ABCG2* gene showed increased resistance to fludarabine (3.5-fold for human *ABCG2* and 6-fold for mouse *Abcg2* in MDCKII transfected cells, P < 0.05) (de Wolf et al., 2008). Similar behavior has been shown for related nucleoside analogs such as clofarabine (Hermann et al., 2021). The interaction between BCRP and nucleoside analogs seems to favor the parent nucleoside over phosphorylated metabolites, as demonstrated by preferential efflux of clofarabine compared to clofarabine monophosphate (Nagai et al., 2011). These data indicate that BCRP is a clinically relevant efflux transporter for fludarabine and its overexpression can compromise treatment efficacy by decreasing intracellular drug levels.

### 6.2 P-glycoprotein

P-glycoprotein (P-gp), encoded by the *ABCB1* gene, is an ATP-binding cassette (ABC) efflux transporter, widely expressed in all barrier tissues and classically involved in multidrug resistance (Hermann et al., 2021; Cole et al., 1992).

For fludarabine, however, both experimental and clinical data indicate that P-gp plays only a minor role in transport (Gerrard et al., 2009; Pallis et al., 2011; Higashi et al., 2000; Malagola et al., 2007; Grey et al., 1999; Svirnovski et al., 2010; Michelutti et al., 1997). In AML studies, P-gp overexpression has been associated with reduced response rates to conventional chemotherapy regimens (Boyer et al., 2019). In addition, fludarabine-based induction protocols, such as FLAG, have demonstrated effectiveness in bypassing P-gp-mediated resistance mechanisms (Pallis et al., 2011; Higashi et al., 2000). In a clinical study enrolling 106 AML patients, 4-year DFS and OS were comparable in patients with high P-gp-protein expression treated with fludarabine (DFS: 28.1%, OS: 33.5%) than for patients not treated with fludarabine (DFS: 33.8%, OS: 46.2%), with no statistically significant differences observed (p = 0.27 and 0.67 for DFS and OS, respectively). These findings conclude that high P-gp protein expression does not negatively impact the efficacy of fludarabine (Malagola et al., 2007). This efficacy is attributed to the fact that fludarabine is not a classical P-gp substrate, allowing it to circumvent traditional multidrug resistance pathways (Gerrard et al., 2009; Grey et al., 1999; Svirnovski et al., 2010; Michelutti et al., 1997). In peripheral blood cells from CLL patients incubated with fludarabine at 25 µg/mL, P-gp protein expression did not correlate with survival status. Moreover, the cytotoxic effect of fludarabine was unaffected by the co-administration of P-gp inhibitors (Grey et al., 1999). Together, these findings support the rationale that fludarabine is not a P-gp substrate.

### 6.3 MRP

The MRP (*ABCC*) subfamily consists of nine related transporters (*ABCC1-6*, *ABCC10-12*) with overlapping but distinct substrate specificities and domain architectures (Wang et al., 2021; Choudhuri and Klaassen, 2006). MRP4 and MRP5, in particular, are established efflux pumps for monophosphorylated nucleoside analogs, while MRP7 and MRP8 have been implicated in resistance to cytarabine, gemcitabine and fluoropyrimidines (Guo et al., 2009; Adema et al., 2014; Russel et al., 2008; Pan et al., 2024; Drenberg et al., 2016; Ritter et al., 2005; Oguri et al., 2012; Hopper-Borge et al., 2009).

Unlike other nucleoside analogs, fludarabine resistance is not mediated by major MRP transporters involved in nucleoside drug efflux. For example, no increase in resistance was observed in cells overexpressing either MRP4 or MRP5, compared with wild-type HEK293 cells (Reid et al., 2003). Clinical data, however, suggest that genetic polymorphism in MRPs may influence outcomes after fludarabine-based conditioning regimens for HSCT. In a study with 85 adult patients with hematological malignancies, receiving a conditioning regimen that included fludarabine in combination with cyclophosphamide and total body irradiation prior to HSCT, the intronic *ABCC4* variant *rs9561778* was associated with increased non-relapse mortality at day 180 (HR = 6.69, 95% CI = 1.83–24.45; P < 0.01) (Takahas et al., 2022). No studies are available on MRP-7 or MRP8-mediated transport of fludarabine. Since several MRPs, including MRP4, are expressed in the blood-brain barrier and renal tubules, variants in these genes could potentially contribute to the neurotoxic effects of fludarabine; however, this remains to be validated (Zhang et al., 2000; van Aubel et al., 2002; Jacobson et al., 2005; Chun et al., 1986; Annaloro et al., 2015; Beitinjaneh et al., 2011). Therefore, current evidence involves MRPs in the modulation of other nucleoside analogs besides fludarabine, although more work is required.

## 7 Conclusion

Fludarabine’s pharmacologic profile is defined by interconnected actions of influx transporters, activating and inactivating enzymes, and efflux pumps that together shape intracellular fludarabine disposition, triphosphate exposure and ultimately, therapeutic response. Among these determinants, dCK-mediated phosphorylation represents the pivotal activating step, while 5′-nucleotidase- and ADA-driven inactivation, SAMHD1-dependent triphosphate hydrolysis, and the activity of specific SLC and ABC transporters (notably hENT1/hCNT3 for uptake and BCRP for efflux) contributed to refine fludarabine disposition at a cellular level. Key enzymes and transporters involved in fludarabine disposition and their supporting evidence are summarized in Table 1. Combining this mechanistic knowledge with PK data, pharmacogenetics, and disease-related changes in transporter and enzyme expression could provide a rational framework for identifying potential biomarkers to integrate in model-informed precision dosing of fludarabine. This would enable future individualized dosing strategies and combination approaches, with the goal of optimizing the benefit–risk balance of fludarabine in HSCT and CAR-T settings, as well as in other hematology malignant therapies.

**TABLE 1:** Synthetic table for fludarabine cellular pharmacology.

Enzyme/transporter	Gene	Function	Tissue expression	Relevance for fludarabine pharmacological pathway	References of function and tissue expression	References for *in vitro* evidence	References for *in vivo*/*ex vivo* evidence
Equilibrative nucleoside transporter 1 (hENT1)	*SLC29A1*	Bidirectional, sodium-independent nucleoside transporter at the plasma membrane	Hematopoietic, epithelial, and endothelial cells	+	Pastor-Anglada et al. (2004), Kaur et al. (2022)	Molina-Arcas et al. (2005), Sirotnak and Barrueco (1987), Griffiths et al. (1997), King et al. (2006), Yamauchi et al. (2014), Dumontet et al. (1999), Lang et al. (2001)	Mackey et al. (2005), López-Guerra et al. (2008), Galmarini et al. (2002), Chow et al. (2005), Gam et al., (2003), Fernández Calotti et al. (2008)
Equilibrative nucleoside transporter 2 (hENT2)	*SLC29A2*	Bidirectional nucleoside transporter with broader substrate range and different inhibitor sensitivity	Widely expressed with high levels in skeletal and smooth muscle	+/−	Pastor-Anglada et al. (2004), Kaur et al. (2022), Ward et al. (2000), Young et al. (2013)	Molina-Arcas et al. (2005), Molina-Arcas et al. (2003), Lang et al. (2004), Lang et al. (2001), Elwi et al. (2009b)	Mackey et al. (2005), López-Guerra et al. (2008)
Concentrative nucleoside transporter 1 (hCNT1)	*SLC28A1*	Sodium concentrative transport	Kidney, liver, small intestine. subsets of leukemic cells	−	Pastor-Anglada et al. (2004), Kaur et al. (2022), Young et al. (2013)	Lang et al. (2004)	​
Concentrative nucleoside transporter 3 (hCNT3)	*SLC28A3*	Sodium-dependent, concentrative transporter for purine and pyrimidine nucleosides	Ubiquitous, most abundant in mammary gland, pancreas, bone marrow, trachea and intestine. Present in Hematopoietic cells	+	Kaur et al. (2022), Young et al. (2013), Pastor-Anglada and Pérez-Torras (2018), Tsang et al. (2008)	Elwi et al. (2009a), Ritzel et al. (2001), Elwi et al. (2009b), Fernández-Calotti and Pastor-Anglada (2010)	Mackey et al. (2005), Fernández-Calotti et al. (2012), Song et al. (2015), Tsang et al. (2008)
Ergothioneine transporter (ETT)	*SLC22A4*	​	Highly expressed in bone marrow, erythroid precursors, monocytes, kidney, and intestinal tissues	+/−	Galluccio et al. (2024), Gründemann et al. (2005), Galluccio et al. (2024), Gründemann et al. (2022)	Müller and Gründemann (2022), Buelow et al. (2021), Tschirka et al. (2018)	Drenberg et al. (2017), Buelow et al. (2021)
Deoxycytidine kinase	*DCK*	Cytosolic deoxynucleoside kinase that phosphorylates purine and pyrimidine nucleosides to monophosphates	Lymphoid tissues and lymphocytes. Low in liver, kidney and pancreas	+	Shu et al. (2010), Arnér and Eriksson (1995), Eriksson et al. (2002), Radu et al. (2008), Eriksson et al. (1994), Spasokoukotskaja et al. (1995), Hatzis et al. (1998)	Brockman et al. (1977), Kemena et al. (1992), Månsson et al. (1999), Yamamoto et al. (2007), Hapke et al. (1996), Tseng et al. (1982), Shewach et al. (1992), Dumontet et al. (1999), Bai et al. (1998), Spasokoukotskaja et al. (1999)	Mackey et al. (2005), Klanova et al. (2014), Rivero et al. (2011)
Deoxyguanosine kinase	*DGUOK*	Mitochondrial deoxynucleoside kinase that phosphorylates purine deoxynucleosides to maintain mitochondrial DNA	Ubiquitous Highly expressed muscle, brain, liver, and lymphoid tissues	−	Johansson and Karlsson (1996)	Månsson et al. (1999), Spasokoukotskaja et al. (1999), Sjöberg et al. (1998)	​
Cytosolic 5′-nucleotidase II (cN-II)	*NT5C2*	Cytosolic 5′-nucleotidase that dephosphorylates IMP, AMP and some nucleoside monophosphates	Ubiquitous Liver, heart, hematopoietic cells	+	Walldén et al. (2007), Ipata and Tozzi (2006)	Yamauchi et al. (2014), Dumontet et al. (1999), Jordheim et al. (2006), Cividini et al. (2015)	Mackey et al. (2005)
Ecto-5′-nucleotidase (CD73)	*NT5E*	GPI-anchored ecto-5′-nucleotidase converts extracellular AMP to adenosine	Widely expressed on epithelial and endothelial cells, fibroblasts and lymphocyte subsets	+	Minor et al. (2019), Resta et al. (1998)	Galmarini et al. (2002), Gam et al. (2003)	Mohanan et al. (2017), Mackey et al. (2005)
Adenylate kinase (AK)	*AK*	Cytosolic enzyme catalyzing the reversible transfer of the γ-phosphate from ATP to AMP	Ubiquitous	+/−	Dzeja and Terzic (2009)	Fi et al. (2018)	​
Nucleoside diphosphate kinase (NDPK1/2)	*NME1/2*	Cytosolic enzyme catalyzing the reversible transfer of the γ-phosphate from nucleoside triphosphate to nucleoside diphosphate	Ubiquitous	+	Schaertl et al. (1998), The Human Protein Atlas (2025b)	​	Braunagel et al. (2012), Zhu et al. (2018)
Cytidine monophosphate kinase 1 (CMPK1)	*CMPK1*	Cytosolic enzyme catalyzing the phosphorylation of pyrimidine nucleosides monophosphates	Ubiquitous	−	Ryu et al. (2011), The Human Protein Atlas (2025a)	Topalis et al. (2016)	​
Sterile alpha motif and histidine-aspartic domain-containing protein 1 (SAMHD1)	*SAMHD1*	Nuclear enzyme hydrolyzing nucleoside triphosphate into nucleoside and inorganic triphosphates	Highly expressed in monocytes, macrophages, dendritic cells, and resting T cells	+	Johansson et al. (2018), Knecht et al. (2018)	Johansson et al. (2018)	Zhu et al. (2018), Schneider et al. (2017), Roider et al. (2021)
Adenosine deaminase (ADA)	*ADA1* *ADA2*	Cytosolic and extracellular deaminase converting adenosine and deoxyadenosine to inosine and deoxyinosine	Highly expressed in the lymphoid organs, such as lymph nodes, spleen, and thymus	+	Bagheri et al. (2019), Paul et al. (2017), Valerio et al. (1985)	Brockman et al. (1977), Avramis et al. (1989)	Noker et al. (1983), Yin et al. (2010)
Cytochromes P450	*CYP450* (e.g., *CYP4502D6*)	Phase I enzymes oxidizing endogenous and exogenous substances	Highly in liver and intestine, present in kidney, lung and brain	−	Gilani and Cassagnol (2025)	​	Johnson et al. (2013)
UDP-glucuronosyltransferase (UGT) 1A4; UGT2B17	*UGT1A4* *UGT2B17*	Phase II enzyme glucuronidating xenobiotics and endogenous substrates	Liver bile conducts, colon and small intestine. High UGT2B17 expression in lymphoid tissue	UGT1A4 ± UGT2B17 +	Allain et al. (2020a), Gruber et al. (2013), Benoit-Biancamano et al. (2009)	Allain et al. (2020a)	Allain et al. (2020a), Gruber et al. (2013)
ABC efflux transporter BCRP	*ABCG2*	ATP-dependent efflux pump for diverse xenobiotic agents	Highly expressed in intestinal epithelium, hepatocytes, renal proximal tubules, placenta and the blood–brain barrier	+	Robey et al. (2009), Doyle et al. (1998)	de Wolf et al. (2008)	Damiani et al. (2010)
ABC efflux transporter P-gp	*ABCB1*	ATP-dependent efflux pump for diverse xenobiotic agents	Ubiquitous	−	Hermann et al. (2021), Cole et al. (1992)	Dumontet et al. (1999), Michelutti et al. (1997), Zeng et al. (2004)	Russo et al. (2005), Damiani et al. (2010), Pallis et al. (2011), Higashi et al. (2000), Malagola et al. (2007), Grey et al. (1999), Svirnovski et al. (2010)
ABC efflux transporter MRPs	*ABCCs*	Multidrug resistance protein transporting nucleotide analogues and cyclic nucleotides	Widely expressed in barrier and excretory tissues (liver, intestine, kidney, lung, brain barriers, placenta, skin) High expression of ABCC1 and ABCC4 in hematopoietic cells	+/−	Zhang et al. (2000), van Aubel et al. (2002), Flens et al. (1996), Tang et al. (2010)	Hopper-Borge et al. (2009), Reid et al. (2003), Guo et al. (2003)	Guo et al. (2009), Takahas et al. (2022)
